# Optical Target Projector: Principle of Functioning and Basic Performance Test

**DOI:** 10.3390/s24175728

**Published:** 2024-09-03

**Authors:** Junzhen Meng, Yabing Xuan, Guiping Huang

**Affiliations:** College of Surveying and Geo-Informatics, North China University of Water Resources and Electric Power, Zhengzhou 450000, China; mengjunzhen@ncwu.edu.cn

**Keywords:** industrial photogrammetry, optical target point, measurement repeatability, surface measurement precision, comparison test

## Abstract

Faced with measurement conditions such as high-temperature forging, strict prohibition of surface contamination, and toxic environments, using the projection point of an optical target projector (referred to as an “optical projector”) as a photogrammetric target has become a necessary method of high-precision industrial photogrammetry. In connection with the current industrial demand, we have analyzed the principles of optical projectors and introduced their optical characteristics and advantages in the field of industrial photogrammetry. On this basis, a series of tests such as brightness, roundness, and so on were conducted to determine the basic properties of the optical projector. A set of performance test methods including inner coincidence accuracy and outer coincidence accuracy were proposed; the tests included industrial photogrammetry system measurement repeatability, surface measurement precision, and a comparison test with laser tracker. The test conditions used optical projection points as the photogrammetry targets. The test results showed that the coordinate measurement repeatability of the industrial photogrammetry system is 0.010 mm, and the surface measurement precision is 0.007 mm under the condition of a single optical projector station, with little difference between the results under the condition of pasting retro-reflective targets. In the process of the comparison test with laser tracker, the image quality of the black measurement object obtained is obviously inferior to other surfaces, so the analysis of the point projector is greatly affected by the color of the measured object and other conditions, which provides a reference for the measurement object and application range of the industrial photogrammetric system based on optical targets. The results demonstrate the applicability and reliability of using the optical projection point of an optical projector as target points for photogrammetry.

## 1. Introduction

The industrial photogrammetric system acquires more than two digital images of the same object in different positions and directions, and then obtains the precise three-dimensional coordinates of the measured points after automatic processing, such as computer feature extraction, image matching, and related mathematical calculations [1,2]. Therefore, the fundamental technology of industrial photogrammetric systems is to produce high-quality “quasi-binary images” [3]. This requires the features of the object to be measured to stand out from their surroundings. Most measured objects lack obvious and easily identifiable feature points or high-contrast features, so it is necessary to lay out obvious measurable targets on the measured objects in order to perform high-precision industrial photogrammetry work [4,5].

Commonly used photographic targets are divided into two types based on their optical characteristics: Retro-Reflective Targets [6,7] and optical targets [8]. During the measurement process, the reflective target point must adhere to the surface of the measured object. However, as industrial photogrammetry becomes more widely used, the measurement objects become more diverse. For some measurement objects, such as high-temperature forgings, toxic, or heavily polluted work pieces, reflective targets cannot be pasted to the surface, emphasizing the benefits of non-contact optical targets. Optical targets are typically projected onto the surface of the measured object using an optical point projector [9,10]. The projected optical targets are extremely bright and can produce a significant contrast with the surface of the measured object. The geometrical size and surface features of the measured object can be precisely measured using projected targets [10,11,12,13].

**Figure 1:**
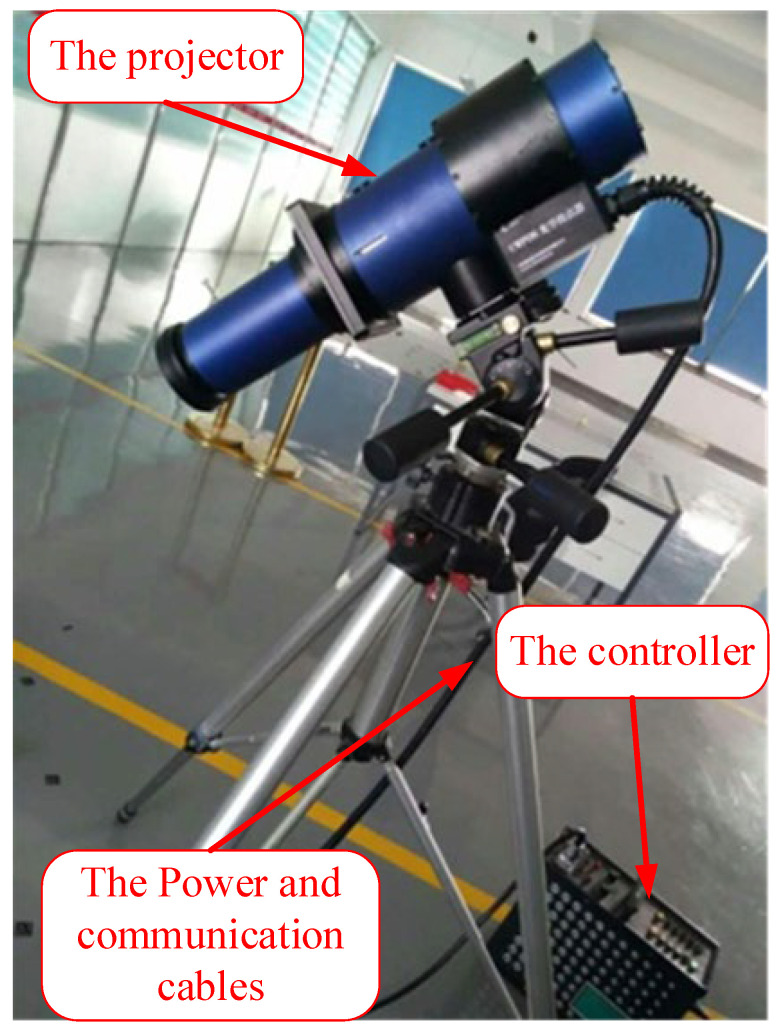
Installation diagram of the optical projector.

Internationally, research and application of optical dowels began earlier, as evidenced by the PRO-SPOT optical dowel from GSI Company in the United States. However, in China, the development of optical target projectors has only recently begun, as exemplified by the independent research and development of an MPS industrial photogrammetry system by Zhengzhou Chenway Technology Co., Ltd., Zhengzhou, China. based on the development of a supporting CWPOS optical target projector (Figure 1). However, there have been few studies on the performance and applicability of the optical target projector, and measurements are often based on experience [14,15]. Using the optical point projector, this paper conducts a series of experiments, including a measurement repeatability test of an industrial photogrammetry system(the version number of software: MPS 4.0.0) based on an optical point projector and a shape and surface measurement accuracy test, to provide a reference for practical measurement engineering. The advantages of the optical projector combined with the industrial photogrammetry system, such as high precision, simplicity, speed, and non-contact, are then demonstrated through practical application cases [15,16].

## 2. Principle and Composition

**Figure 2:**
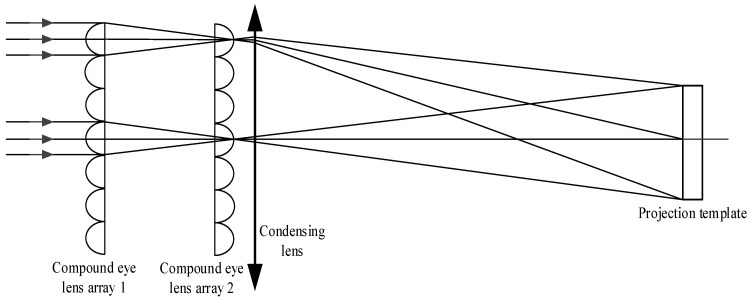
Principle diagram of the optical target projector.

The imaging principle of convex lenses serves as the foundation for the projector (as illustrated in Figure 2). The light emitted by the modeling lamp and the flash is refracted through a series of optical elements, including the condensing lens and the uniform optical device, resulting in significantly improved uniformity. Finally, the light passes through the corresponding aperture holes on the projection template and a set of convex lenses, forming a highlighted point on the surface of the measured object [17,18]. Among them, the uniform optical device uses a compound eye lens, which is made up of a series of small lenses [15,16]. The use of a double-row compound eye lens array can result in high light energy utilization and uniform illumination over a large area.

**Figure 3:**
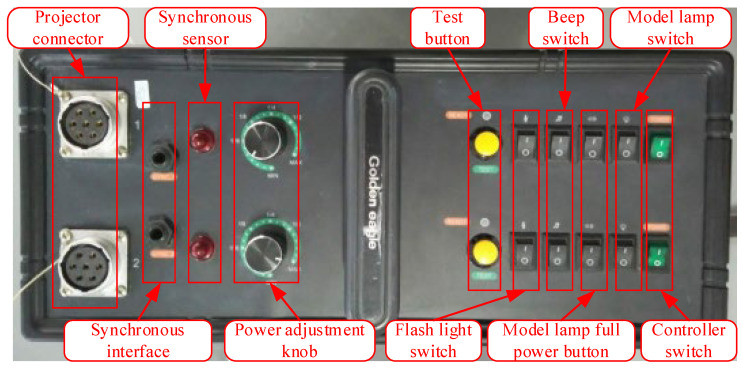
Operator interface of the Optical Target Projector.

In principle, an optical projector consists of two parts: the projector and the controller, which are connected by a cable (Figure 1). The projector consists of various components, including a control circuit board, reflective bowl, modeling lamp, spiral flash lamp, condenser lens, uniform optical device, and projection template. The controller consists of a power supply component and a projector control panel, which are used to configure the projector’s power parameters and power supply (Figure 3). Typically, the optical point feeder is equipped with a number of different points of templates that can be selected based on the measurement requirements in practical applications [19,20,21].

## 3. Features and Advantages

The characteristics of the throwing device are readily apparent from an examination of the throwing principle of the optical target projector.

High efficiency: a complete measurement in a few minutes with a single camera.

Adjustable point cloud density: can project hundreds to thousands of points at a time, with template design and customization as required.

Flexible layout: the projector is set up on a tripod, which can be adjusted depending on the measurement object.

High contrast: the brightness of the projection point is very high, creating a high brightness contrast with the background, which is conducive to the recognition of feature points.

Durability: the system is robust and can be used in a variety of measurement environments.

Based on the above-mentioned characteristics, the point projector has many advantages in practical applications: First and foremost, the optical point projector is used to achieve true non-contact measurement of the surface of the measured object, providing the measurement basis and conditions for the measurement of high-temperature, toxic, and other unconventional objects, thereby expanding the application field of industrial photogrammetry. Secondly, the use of a point projector eliminates the effect of reflective target thickness on the measurement results [22]. When the shape surface is measured, the effect of uneven target thickness on the fitting results is eliminated. Finally, the layout of the target projector is flexible, allowing for multiple splice measurements and encryption measurements of key parts as needed, meeting a variety of measurement requirements and increasing the applicability of industrial photogrammetry.

## 4. Basic Performance Testing and Analysis

The brightness and roundness of the optical target points are important factors affecting the measurement accuracy, so the basic research on the performance of the optical point projector is mainly concerned with experimentally testing whether the brightness and roundness of the projection point of the optical point projector meet the measurement requirements.

### 4.1. Projection Point Brightness

**Figure 4:**
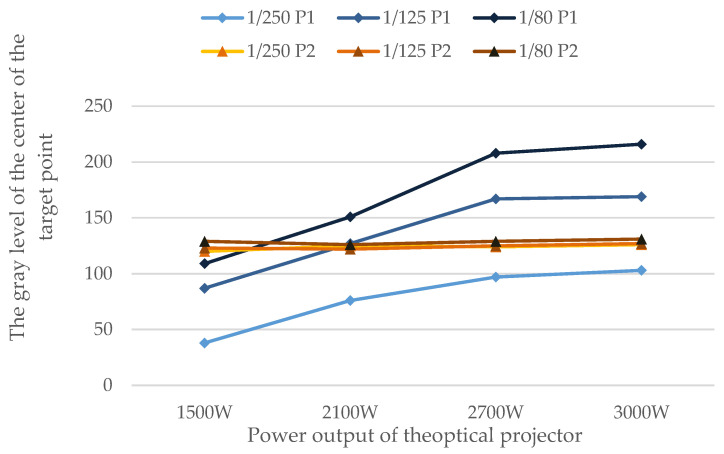
Test results analysis diagram of the output power of the projector flash effect on the projection target gray level.

The brightness of the projection point of the optical target is primarily determined by the brightness of the projector’s flash, but it is also affected by the size of the aperture of the lens of the projector. The brightness of the optical target points is directly reflected in the gray value of the obtained image mark points. The test involves adjusting the output power and aperture size of the point projector’s flash, taking photos of the projected targets under various conditions, processing the images, obtaining the gray value, and comparing them to reflective target points. The gray values of the optical target point (P1) and the directional reflective target point (P2) in the central region were compared, and the results are as Table 1:

Figure 4 shows that as the output power of the flash increases, the gray level of the projected targets increases, and as the shutter speed slows, the exposure time increases, as does the gray value of the targets in the resulting image. When the output power of the projector is 2100 W and the shutter speed of the camera is 1/125, the gray level of the optical target at the center of the projection area is approximately equal to that of the directional reflective target.

Based on this experiment, the output power of the dowel is kept at 2100 W while the aperture size is changed. The aperture value of 1 indicates that the dowel is fully open. The following is roughly divided into 1/2, 1/3, and 1/4 based on the aperture radius.

**Figure 5:**
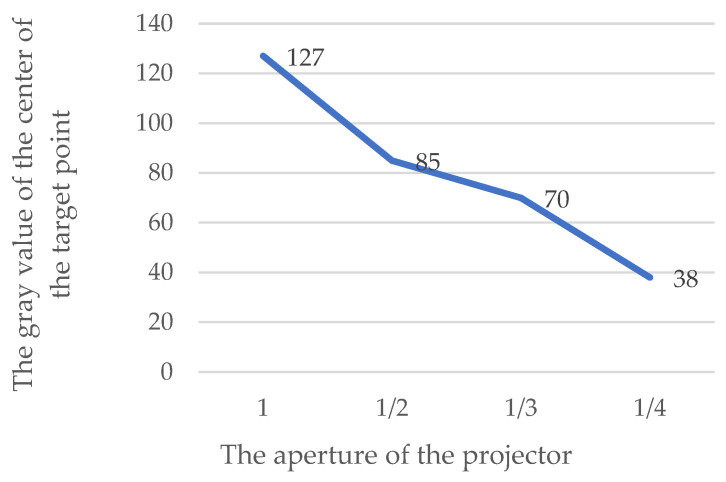
Test results analysis diagram of the projector aperture size effect on projection target brightness.

As shown in Table 2 and Figure 5, the larger the aperture of the projector, the brighter the projected targets.

In conclusion, the optical targets projected by the point projector under appropriate parameters can form a high-brightness contrast with the background and are well recognized by industrial photogrammetry software (MPS 4.0.0), indicating their applicability in the field of industrial photogrammetry.

### 4.2. Circularity of the Projection Point

The premise of industrial photogrammetry is that image recognition can determine the center of the target point, and industrial photogrammetry software can extract the edge of the target point using an edge extraction algorithm, after which the center of the target point can be determined. Therefore, the recognition of projection targets is influenced by both their brightness and their roundness. The following tests are performed to determine whether the projected optical targets can be accurately recognized.

**Figure 6:**
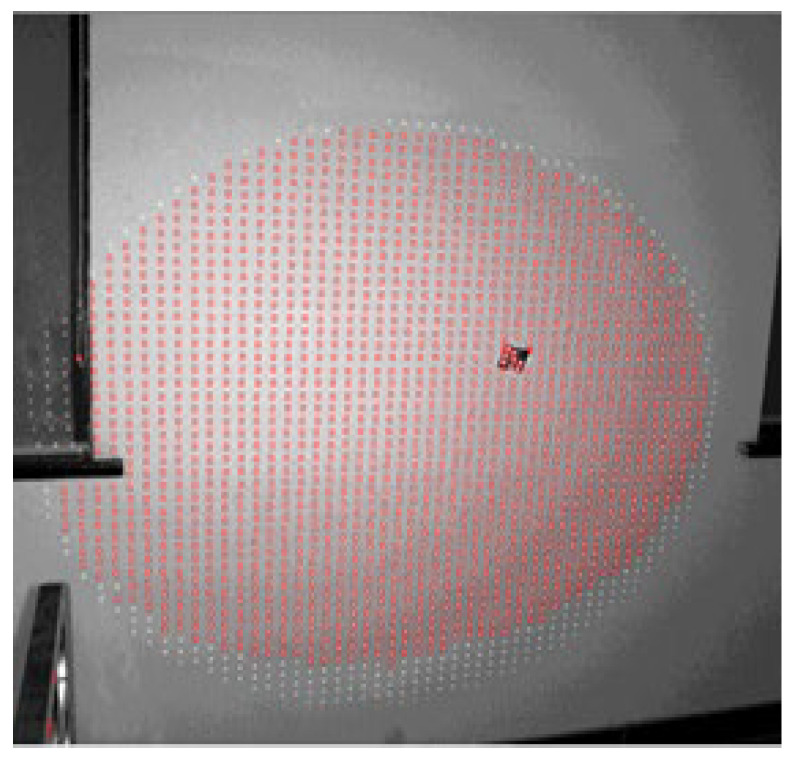
Scan results.

**Figure 7:**
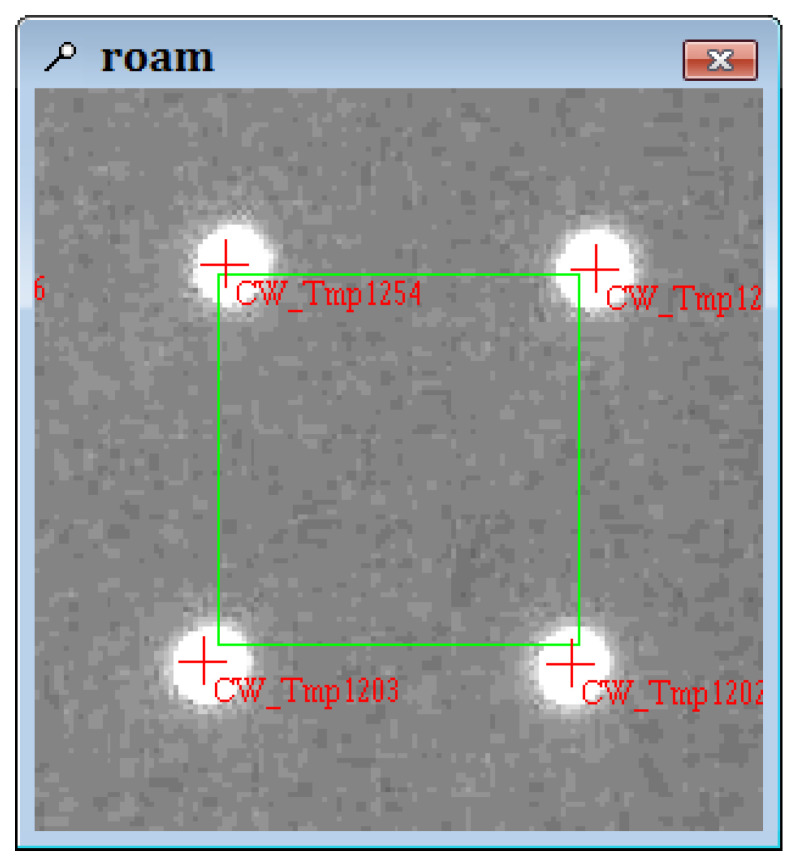
Roundness of optical targets.

**Figure 8:**
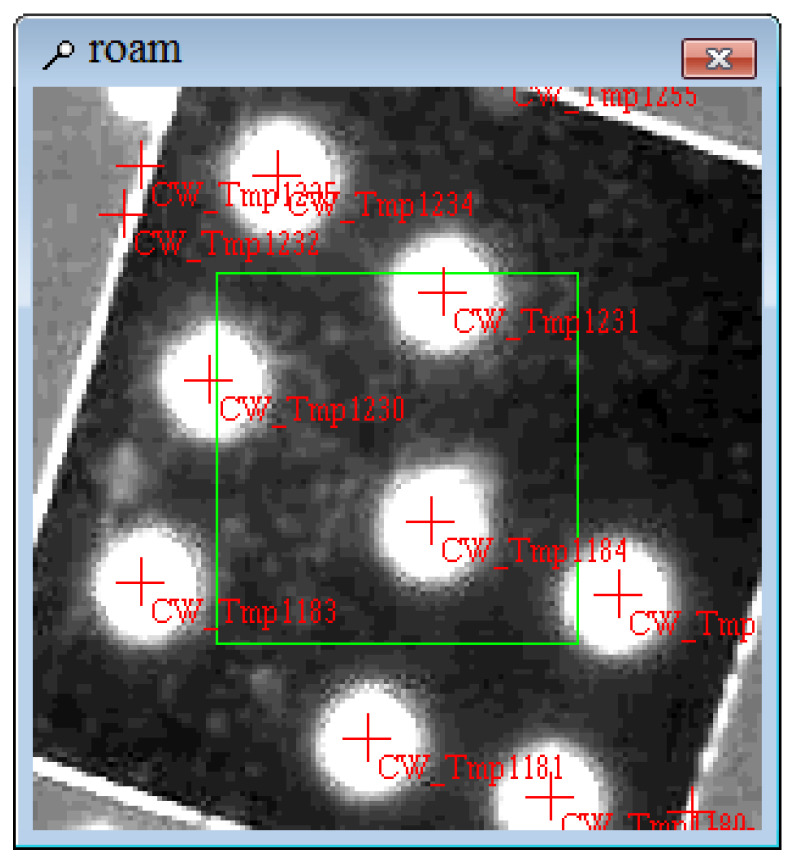
Roundness of directional reflective target points.

According to the results of the brightness test, the parameters of the projector are adjusted: the aperture is set to maximum, the projection distance is 1.9 m, and the output power is modulated to 2100 W. The image of the projected target point is obtained and scanned by the target, and the roundness of the optical target is compared with the reflection target of the backlight. Figure 6, Figure 7 and Figure 8 show the experimental results.

As shown in Figure 6, the brightness of the projector increases when projected onto a white wall, and the projection effect improves. When scanning conditions such as brightness, roundness, and overexposure are used as scanning conditions, the scanning range remains large, and the projected optical targets can be accurately identified. Comparing Figure 7 and Figure 8, the circularity of the projection point and the reflective target point are similar, and both are good, indicating that optical target points have industrial applications.

## 5. Accuracy Test

In this paper, a series of test analyses were carried out from two aspects of accuracy and precision to verify the stability of the optical point projector during the point projection process and the measurement accuracy of the MPS industrial photogrammetry system based on the point projector when projecting optical targets. These tests included a repeatability test, a shape measurement accuracy test, and a comparison test with a laser tracker. Among them, the repeatability test results compared with retro-reflective targets of already mature application, and we analyzed the applicability and reliability of the projector based on this.

### 5.1. Repeatability Test

Repeatability refers to the accuracy with which the same quantity is observed multiple times, indicating the repeatability and stability of the system [23]. The targets are measured repeatedly according to the same measurement network shape, and then the coordinates of the measured object points are converted to common points. In common point conversion, the coordinates of the landmarks in the first group are generally used as the basis for common point conversion. Finally, the RMS of all the landmarks measured by the two groups is calculated according to the difference in the coordinates of the same landmarks. The RMS value reflects the dispersion of multiple measurement results, so as to reflect the stability of the camera [24].

(1) $$RMS_{i1}\_X=\sqrt{\frac{{\left(X_{i1}-X_{11}\right)}^{2}+{\left(X_{i2}-X_{12}\right)}^{2}+\cdots +{\left(X_{in}-X_{1n}\right)}^{2}}{n-1}}$$

(2) $$RMS_{i1}\_Y=\sqrt{\frac{{\left(Y_{i1}-Y_{11}\right)}^{2}+{\left(Y_{i2}-Y_{12}\right)}^{2}+\cdots +{\left(Y_{in}-Y_{1n}\right)}^{2}}{n-1}}$$

(3) $$RMS_{i1}\_Z=\sqrt{\frac{{\left(Z_{i1}-Z_{11}\right)}^{2}+{\left(Z_{i2}-Z_{12}\right)}^{2}+\cdots +{\left(Z_{in}-Z_{1n}\right)}^{2}}{n-1}}$$

(4) $$RMS_{i1}\_P=\sqrt{RMS_{i1}\_X^{2}+RMS_{i1}\_Y^{2}+RMS_{i1}\_Z^{2}}$$

(5) $$average=\frac{RMS_{21}+RMS_{31}+\cdots +RMS_{m1}}{m-1}$$

(*X_ij_*, *Y_ij_*, *Z_ij_*) is the coordinate value of the mark point, *i* = 1, 2… *m*; *j* = 1, 2… *n*, *m* is the number of measurements; *n* is the number of points with the same name; and *RMS_i_*_1__*X*, *RMS_i_*_1__*Y*, and *RMS_i_*_1__*Z* represent the root-mean-square error of group *i* coordinate points in *X*, *Y*, and *Z* directions, respectively.

The repeatability of the MPS industrial photogrammetry system has been extensively tested and verified using reflective targets [25,26]. Using this as a reference, the point coordinate measurement repeatability of the MPS industrial photogrammetry system is tested when targets are projected using a point projector, and the repeatability and stability of the point projector and MPS industrial photogrammetry system are analyzed. The test process is as follows.

#### 5.1.1. Layout of the Test Site

**Figure 9:**
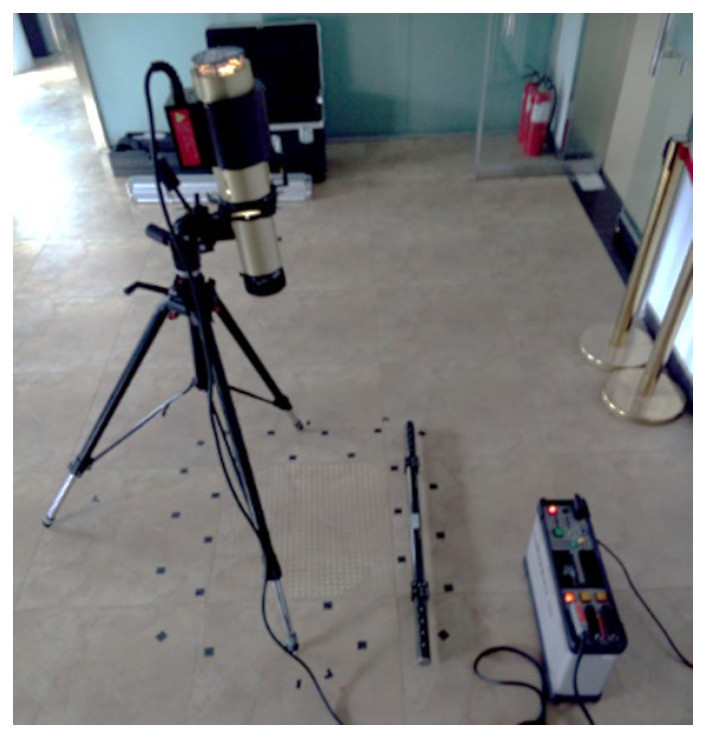
Layout of the test site.

A tile with a relatively flat area of 600 mm × 600 mm is selected as the measurement object, and the coded target points are evenly distributed within a 200 mm wide range around it to complete the test site layout (Figure 9).

#### 5.1.2. Setting of Test Parameters

In addition to optimizing the projector’s height, aperture size, and flash intensity at each position, it is also necessary to optimize the camera’s shooting parameters, including exposure time, flash intensity, and so on. The optimal parameters are determined based on the brightness of the targets and background in the resulting images. Table 3 shows the parameter settings after optimization.

#### 5.1.3. Repetitive Test

**Figure 10:**
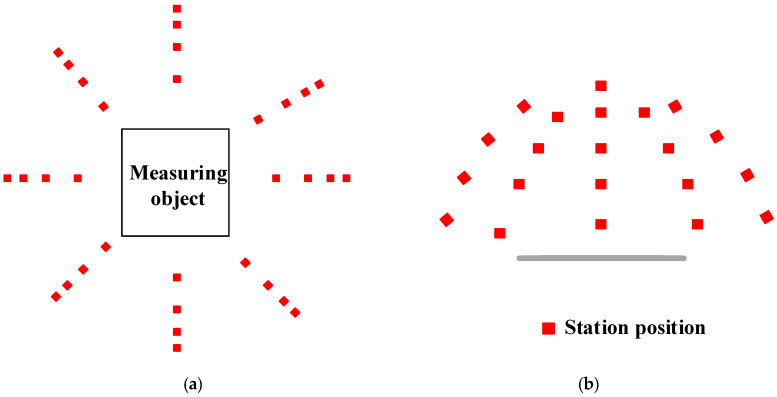
Measurement network diagram: (**a**) Top view of measurement network; (**b**) Side view of measurement network.

**Figure 11:**
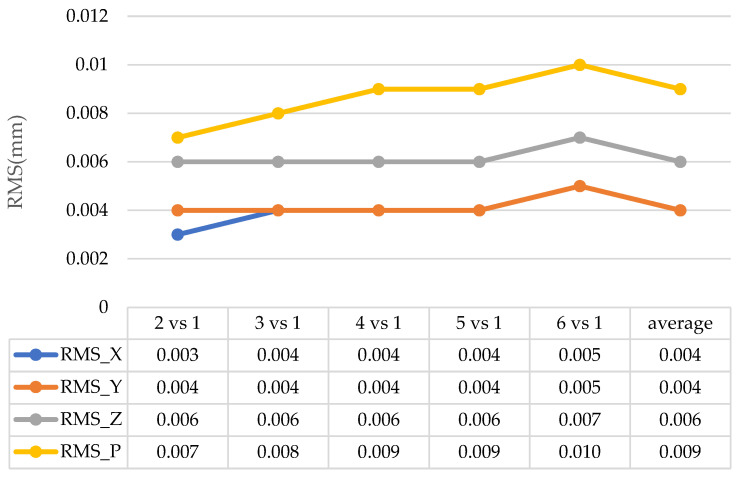
Repeatability test results for optical target measurement.

**Figure 12:**
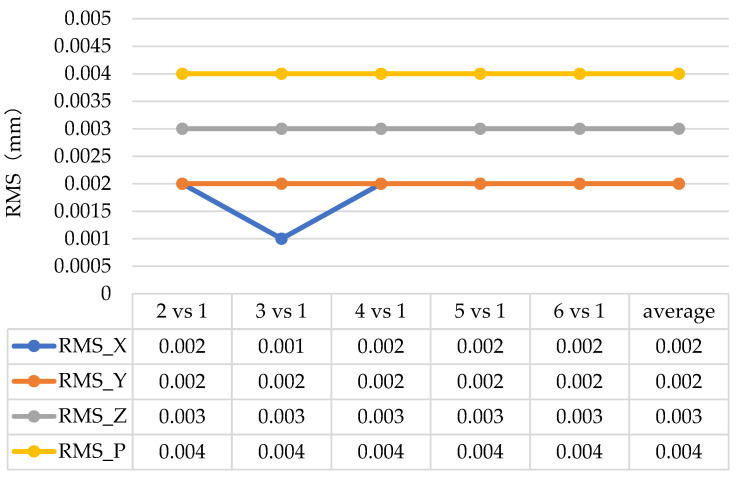
Paste reflective target points repeatability test results.

The repeatability test was divided into two parts. In the first part, assuming that the parameter settings were completed, the point projector projected the target points on the surface of the plane and the entire plane at once. The CIM-2+ industrial measuring camera was used to capture images of the plane with a suitable measuring network shape (Figure 10) while keeping the point projector stable. In the second step, target points were placed on the same measured surface and photographed with a CIM-2+ industrial camera using the same mesh shape as in the first step, as well as six consecutive shots. Finally, the acquired images were processed and calculated using the MPS/S industrial photogrammetry system, yielding the following results:

According to the data in Figure 11 and Figure 12, the average repeatability of the system is 0.009 mm, with a maximum of 0.010 mm when using the optical target projector. When using reflective targets, the system has an average repeatability of 0.004 mm. The repeatability of the point position measurement results of the point projector is slightly lower than that of the sticking target points, but the results can still reach the micron level, which meets the measurement requirements, indicating that the projection point of the optical point projector is highly reliable as a photogrammetric target.

### 5.2. Accuracy Test for Shape Surface Measurements

Shape surface measurement accuracy reflects the accuracy of the system, which is generally measured by the shape surface deviation. The shape surface deviation is an important parameter used to measure the difference between the measurement point and the model value of the measurement surface. The smaller the actual shape surface deviation, the higher the shape surface measurement accuracy.

**Figure 13:**
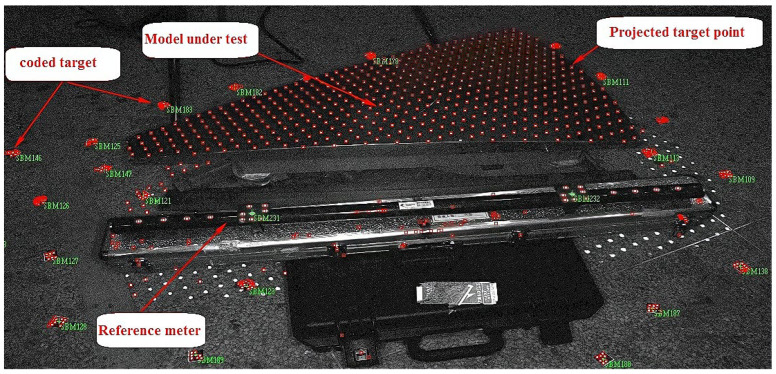
Site layout diagram of a high-precision mold.

A high-precision mold is used as the test object for the shape surface measurement accuracy test (Figure 13). The mold has a sector-curved surface and measures approximately 980 mm in length, 75 mm in width on the short side, and 560 mm in width on the long side. The coordinate measuring machine accurately measured the shape surface of the mold, which is 1.7 μm.

#### 5.2.1. Site Layout

The upper (convex) surface of the mold is measured, while the concave surface is fixed to the metal tool, which is then placed on stable ground. An appropriate number of coded target points are evenly distributed outside the 300 mm width range around the mold to ensure that the coded target is not blocked by the mold when shooting from the opposite side, and the appropriate position is marked on the benchmark ruler to complete the test site layout (Figure 13).

#### 5.2.2. Setting of Test Parameters

Optimize the parameters of each projector position, such as projector height, aperture size, and flash intensity, as well as the shooting parameters of the camera, such as exposure time, flash intensity, and so on. The optimal parameters are determined based on the brightness of the targets and background in the resulting images. Table 4 shows the optimized parameter settings.

#### 5.2.3. Test for the Accuracy of Shape Surface Measurements

**Figure 14:**
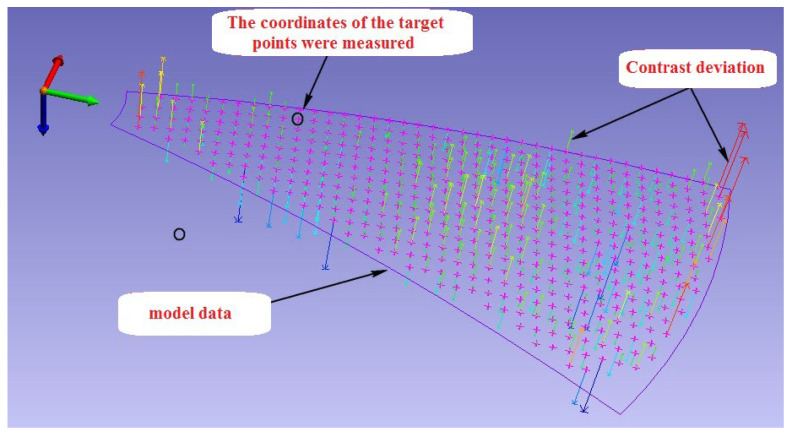
Contrast deviation diagram between measurement and model data.

The surface of the mold was measured with the appropriate mesh shape using the best parameters, and five sets of continuous measurements were made. The measured three-dimensional coordinate values were processed and analyzed based on the measurement repeatability and compared with the model data of the measured mold surface. The shortest distance from the target point to the measured model surface is called the contrast deviation of the feature point from the model (see Figure 14). The root-mean-square of the contrast deviation is called the surface standard deviation, which represents the measurement accuracy of the shape surface.

The statistical analysis of the measurement results is shown in Table 5 and Table 6.

The data in Table 5 and Table 6 show that when the optical projector is used to measure the mold, the point coordinate measurement has a repeatability of 0.010 mm and a measurement accuracy of 0.007 mm for the shape surface. The error of the measurement results in the table should include the measurement error of the industrial photogrammetric system and the error of the standard value. It is known that the shape surface RMS measured by the CMM is 0.0017 mm, far less than the measurement error, so it can be a standard value. According to the law of error propagation, the standard value error is much smaller than the measurement error, so it can be ignored. It can be inferred that the shape surface measurement accuracy of the optical point projector combined with the industrial photogrammetry system is approximately 0.007 mm, indicating that the projection point of the optical point projector as a photogrammetric target can meet the measurement accuracy requirements and has high applicability.

### 5.3. Comparison Test with Laser Tracker

To further verify the accuracy and applicability of the industrial photogrammetry system when using optical targets, a comparative test with the laser tracker was carried out in this paper. The object measured was a marble cube with a length of 60 cm, a width of 60 cm, and a height of 10 cm. The surface of the measured plane is 00 level accuracy (nominal plane fitting RMS is 0.005 mm) marble plane. The object measured was placed on stable ground and was held in place by four supports.

#### 5.3.1. Test Process

**Figure 15:**
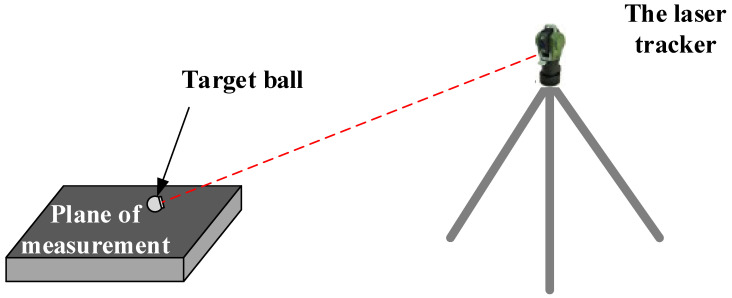
The site layout diagram of laser tracker measurement.

The test consists of two parts. The first part uses a laser tracker to measure the flatness of the marble plane. The sampling modes include single-point measurement and continuous measurement. First, the laser tracker was set up at a distance of 2–3 m from the measured object and connected (as shown in Figure 15). Then, according to the test plan, two modes of single-point measurement and continuous measurement were used respectively to measure, with five groups of single-point measurement and three groups of continuous measurement. Finally, the same fitting method was used for plane fitting of the measured points, and the flatness measuring accuracy and stability of the laser tracker were analyzed.

**Figure 16:**
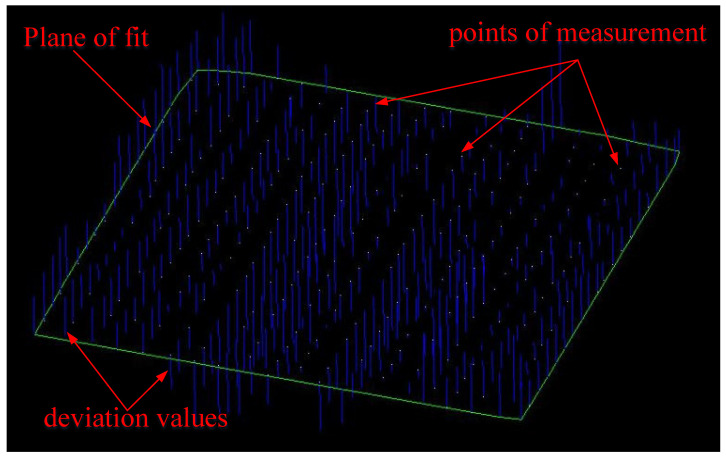
The results of single-point measurement plane fitting.

In the second part, the planarity of the marble plane was measured using an industrial photogrammetry system with the projection target of an optical projector. The projector projects the optical target perpendicular to the measured object. First of all, the height distance, flash intensity, and aperture size of the projector at each position were tested, and the best parameters were determined according to the effect of extracting targets. Then, the marble plane was measured with the appropriate mesh shape (as shown in Figure 16) under the best parameters. Finally, the measurement results were processed and analyzed, and the flatness measurement accuracy of the photogrammetric system with the point projector was evaluated.

#### 5.3.2. Measurement Test of Laser Tracker

According to the conditions of the test site, the laser tracker was set up at a distance of 2–3 m from the measured object at an appropriate angle, as shown in Figure 15. It was then connected to the computer to carry out a single-point or continuous measurement.

When the single-point measurement mode was adopted, the measurement points were evenly distributed according to the size of the measured surface. The specific layout method is as follows: A measurement point was distributed on the measured plane at an average interval of about 3 cm in the vertical and horizontal directions, that is, 400 measurement points were uniformly distributed by the method of 20 mm × 20 mm. The single-point measurement mode of the laser tracker was used to measure these points successively. After all the measurements were completed, the measured point coordinates were exported and imported into the MPS/S software(MPS 4.0.0) for plane fitting (see Figure 16).

**Figure 17:**
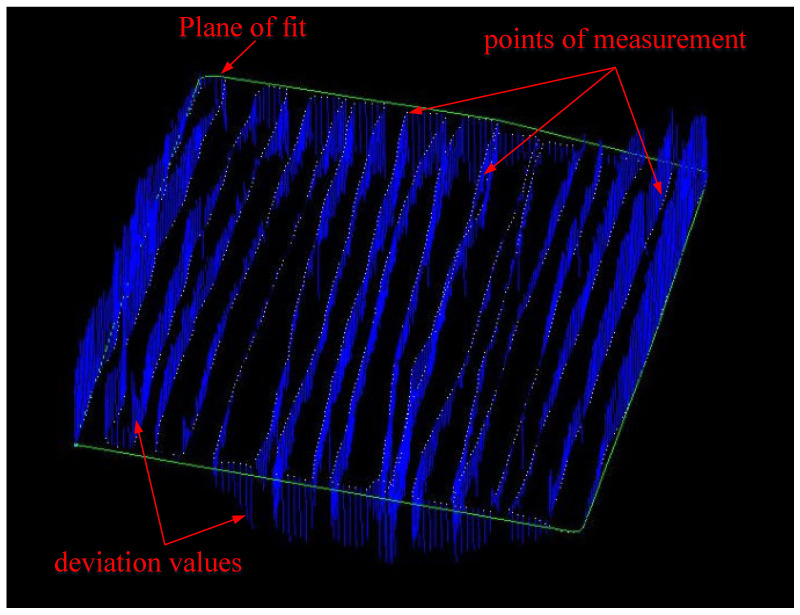
The results of continuous measurement plane fitting.

Continuous measurement means that after the laser tracker starts to measure, the position of the target ball was constantly moved, and the laser tracker tracks and measures the target ball. At this time, the spacing of the measuring points will be small, which can reflect the actual trajectory of the movement (as shown in Figure 17), but the final result is still the three-dimensional coordinates of a single point. These measuring point coordinates were also imported into MPS/S for plane fitting, and the fitting results were analyzed and compared with the single-point measurement results.

#### 5.3.3. Optical Target Measurement Test

**Figure 18:**
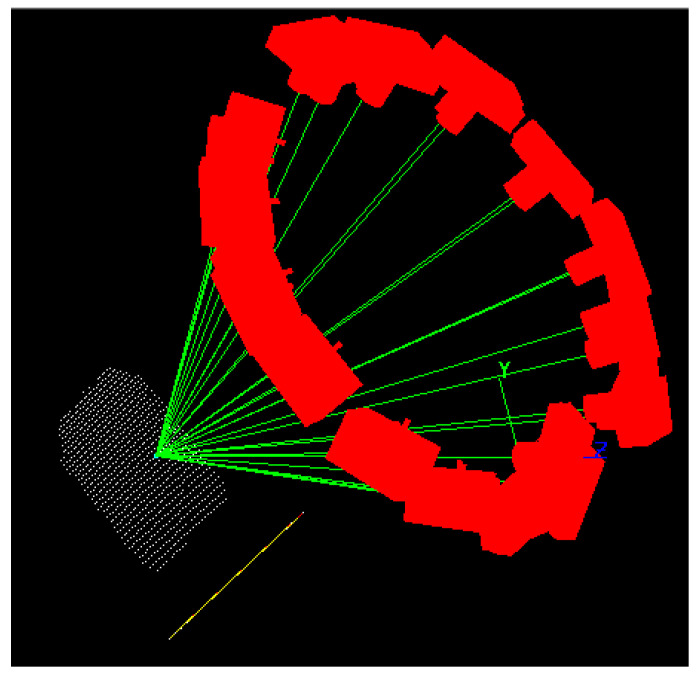
Processing results and camera position figure.

In order to solve the problem and splice the image, it is necessary to arrange the control field evenly around the tested object. The control field was composed of approximately forty 3 mm coded targets arranged evenly and reasonably around the marble platform (as shown in Figure 18). Then, the projector parameters (including projection direction, distance, aperture, and flash brightness) and camera shooting parameters (including time of exposure and flash intensity) were optimized and tested. The optimal parameters were selected according to the brightness of the targets and background in the obtained images. After optimization, the parameter settings were as shown in Table 7.

**Figure 19:**
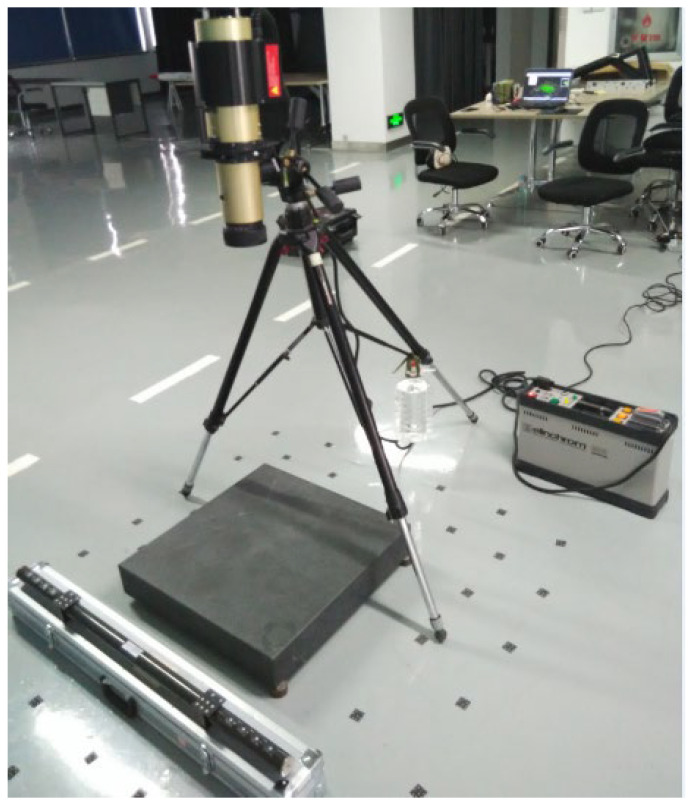
The scene diagram of the measurement site layout.

**Figure 20:**
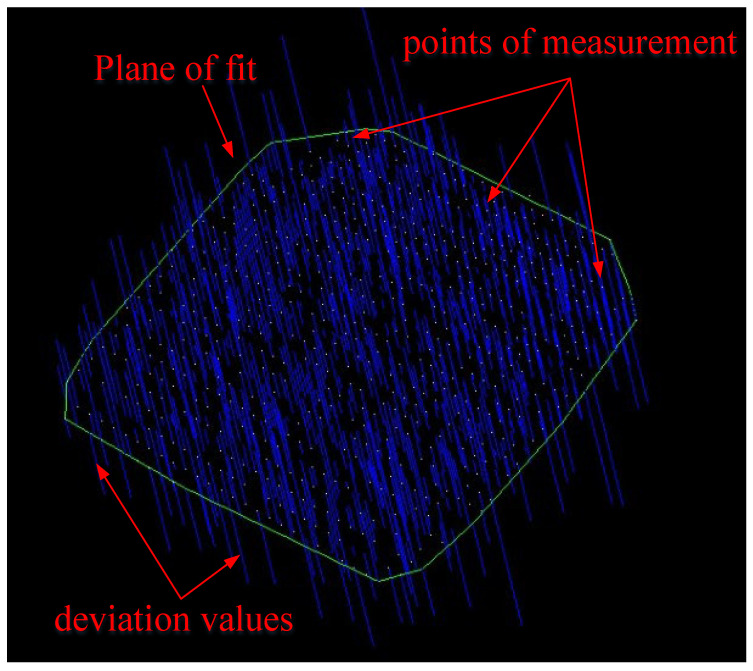
The results of plane fitting of optical target points.

The measurement was carried out according to the optimal parameters selected, and the location of the camera station was evenly distributed around the measured object, as shown in Figure 18. The point feeder was set up perpendicular to the measured plane (as shown in Figure 19), and the measurement results were processed in three groups to calculate their repeatability and plane fitting results (as shown in Figure 20).

#### 5.3.4. Test Results and Analysis

The measurement results of the laser tracker are as follows:

From the analysis of Table 8, it can be seen that (1) the plane fit RMS of the marble plane measured by the laser tracker is 0.010 mm, and the flatness is 0.050 mm, and (2) the ability of single-point measurement is equivalent to that of continuous measurement in flatness measurement, and the stability of single-point measurement is slightly better than that of continuous measurement.

The results of the photogrammetric test using the projector are shown in Table 9.

Table 9 shows that when the projector is used to project the optical target points, the repeatability of the point position coordinate measurement is poor, which is 0.016 mm. The RMS of the plane fit from the measured point position coordinates is 0.035 mm, the flatness is 0.200 mm, and the flatness repeatability is 0.008 mm. Due to the high accuracy of the measured plane (nominal flatness accuracy 0.005 mm), the measured flatness can be obtained by comparing with the laser tracker: when the projector drops the point, the flatness measured by photogrammetry is poor, indicating that its flatness measurement accuracy is not good, but the repeatability is good.

The comprehensive analysis and comparison of the results are as Table 10:

By comparing the flatness measurement results of different measurement methods, the following conclusions can be drawn:

When the laser tracker is used for measurement, the flatness measurement results of single-point measurement and continuous measurement are consistent, both about 0.050 mm, but the stability is not good. When the projector is used to cast points, because the measured object is black and has strong light absorption, the quality of the image obtained is poor (the root-mean-square value of the image point is 0.35 μm, and the normal value is between 0.10 and 0.20 μm), the error of the point position coordinate is large, and the repeatability is not good (0.017 mm). The influence on the final plane fit is large (RMS of plane fit is 0.035 mm), but the repeatability of the flatness measurement is good, which is 0.006 mm, indicating that the influence is systematic. Therefore, it is analyzed that the projection point measurement stability of the projector is good, and it is greatly affected by the color and other conditions of the tested object, which needs further research.

## 6. Conclusions

With different measurement requirements, such as non-contact and high precision, the use of optical projectors in industrial photogrammetry will become more common and receive more attention. Based on an introduction to the principles of optical projectors, this paper examines the benefits of optical projectors and conducts a series of tests on their basic performance. The test results show that the coordinate measurement repeatability of the industrial photogrammetric system is 0.010 mm. This value is comparable to the measurement accuracy of the industrial photogrammetric system when using reflective targets. This suggests that the projection point of the optical projector is highly reliable as a photogrammetric target. Furthermore, the measurement accuracy of the shape surface is 0.007 mm, indicating that the projection point of the optical projector meets the measurement accuracy requirements for a photogrammetric target and has a wide range of application.

However, in the comparative test process with the laser tracker, it was found that the measurement accuracy of the industrial photogrammetry system that uses the projector to project the optical target is significantly reduced. When comparing and analyzing the measurement objects of the three test groups, the image quality of the black measurement object obtained in the comparison test with the laser tracker is significantly inferior to the above two groups. Therefore, the analysis of the projector is greatly affected by the color of the test object and other conditions, which requires further research. The last test provides a reference for the measurement object and application scope of the industrial photogrammetry system based on optical signs. If the surface of the measured object is a color with strong light absorption, such as black, the image quality can be improved by spraying developer, etc. to improve the measurement accuracy.

## Figures and Tables

**Table 1:** Test results of the output power of the projector flash effect on the projection target gray level.

	Camera Shutter(s) Gray Level of a Point	1/250		1/125		1/80	
Power Output of the Optical Projector		P1	P2	P1	P2	P1	P2
1500 W		38	120	87	123	109	129
2100 W		76	125	127	122	151	126
2700 W		97	124	167	125	208	129
3000 W		103	126	169	127	216	131

**Table 2:** Test results of the projector aperture size effect on projection target brightness.

The Aperture of the Projector	1	1/2	1/3	1/4
Central gray value	127	85	70	38

**Table 3:** Repeatability test parameter settings table.

The Optical Projector			
Projection direction	Distance	Aperture	Flash brightness
Vertical	1.26 m	Max	1/3
**Camera**			
Time of exposure		Flash intensity	
5 ms		Lv 3	

**Table 4:** Shape surface parameter settings for measurement accuracy tests.

The Optical Projector			
Projection direction	Distance	Aperture	Flash brightness
Vertical	1.20 m	Max	1
**Camera**			
Time of exposure		Flash intensity	
1/125 ms		1/128	

**Table 5:** Plane fitting measurement results statistics.

Group	Points of Fit	Contour Accuracy RMS (mm)	Maximum Deviation (mm)	Minimal Deviation (mm)
1	469	0.006	0.021	−0.024
2	467	0.007	0.02	−0.025
3	462	0.007	0.024	−0.027
4	467	0.007	0.021	−0.019
5	460	0.007	0.018	−0.034
Average	465	0.007	0.021	−0.026

**Table 6:** Point coordinate measurement repeatability results statistics.

Group	RMS_X (mm)	RMS_Y (mm)	RMS_Z (mm)	RMS_P (mm)
2 vs. 1	0.005	0.005	0.006	0.009
3 vs. 1	0.007	0.005	0.006	0.010
4 vs. 1	0.006	0.005	0.005	0.009
5 vs. 1	0.007	0.006	0.008	0.012
Average	0.006	0.005	0.006	0.010

**Table 7:** Projector and camera parameters settings.

The Optical Projector			
Projection direction	Distance	Aperture	Flash brightness
Vertical	1.20 m	Max	1/3
**Camera**			
Time of exposure		Flash intensity	
5 ms		Level 3	

**Table 8:** Laser tracker flatness measuring results (Units: mm).

	Measuring Results					Repeatability of Flatness
Measurement Pattern	Group	RMS of Plane Fit	Maximum Deviation	Minimal Deviation	Planeness	
Single-point measurement	1	0.012	0.028	−0.036	0.064	0.016
	2	0.015	0.024	−0.044	0.068	
	3	0.006	0.014	−0.016	0.030	
	4	0.009	0.018	−0.025	0.043	
	5	0.012	0.024	−0.032	0.056	
Continuous measurement	1	0.003	0.01	−0.010	0.020	0.023
	2	0.011	0.03	−0.029	0.059	
	3	0.011	0.029	−0.030	0.059	

**Table 9:** Statistical table of measurement results of optical target points.

Statistics of Adjustment Results (mm)					
Group	Image Point Root-Mean-Square Error (μm)	RMS_X	RMS_Y	RMS_Z	RMS of Reference Meter
1	0.34	0.005	0.007	0.012	0.012
2	0.34	0.005	0.007	0.012	0.011
3	0.33	0.006	0.008	0.014	0.016
Average value	0.34	0.005	0.007	0.013	0.013
**Point Coordinate Repeatability Measurement (mm)**					
**Group**	**RMS_X**	**RMS_Y**	**RMS_Z**		**RMS_P**
2 vs. 1	0.007	0.008	0.011		0.015
3 vs. 1	0.008	0.009	0.013		0.017
Average value	0.008	0.009	0.012		0.016
**Planar Fitting of Measurement Results (mm)**					
**Group**	**Number of Fitting Points**	**RMS of Plane Fit**	**Maximum** **Deviation**	**Minimal** **Deviation**	**Planeness**
1	832	0.035	0.101	−0.104	0.205
2	811	0.033	0.098	−0.091	0.189
3	820	0.035	0.1	−0.105	0.205
Average value		0.034	0.100	−0.100	0.200

**Table 10:** The measured results of plane fitting based on different measuring ways (Units: mm).

Measurement Mode	RMS of Plane Fit	Maximum Deviation	Minimal Deviation	Planeness	Repeatability of Flatness
Laser tracker single-point measurement	0.011	0.022	−0.031	0.052	0.016
Continuous measurement by laser tracker	0.010	0.023	−0.026	0.049	0.023
The optical target	0.034	0.100	−0.100	0.200	0.005

## Data Availability

Data are contained within the article.
